# Switch to Dolutegravir plus Rilpivirine Dual Therapy in cART-Experienced Subjects: An Observational Cohort

**DOI:** 10.1371/journal.pone.0164753

**Published:** 2016-10-14

**Authors:** Amedeo F. Capetti, Gaetana Sterrantino, Maria Vittoria Cossu, GianCarlo Orofino, Giorgio Barbarini, Giuseppe V. De Socio, Simona Di Giambenedetto, Antonio Di Biagio, Benedetto M. Celesia, Barbara Argenteri, Giuliano Rizzardini

**Affiliations:** 1 1st Division of Infectious Diseases, ASST Fatebenefratelli-Sacco, Milano, Italy; 2 Division of Infectious Diseases, “Careggi”, Hospital, Firenze, Italy; 3 Division of Infectious Diseases, “Amedeo di Savoia” Hospital, Torino, Italy; 4 2nd Division of Infectious Diseases, “Policlinico San Matteo” Hospital, Pavia, Italy; 5 Infectious Diseases Clinic, Azienda Ospedaliero-Universitaria di Perugia, Perugia, Italy; 6 2nd Division of Infectious Diseases, “Policlinico Universitario Agostino Gemelli” Hospital, Roma, Italy; 7 Infectious Diseases Clinic, “San Martino” Hospital, Genova, Italy; 8 Infectious Diseases Unit, University of Catania, ARNAS (Azienda Ospedaliera di Rilievo Nazionale e di Alta Specializzazione) Garibaldi, Catania, Italy; 9 Whitwaterstrand University, Johannesburg, South Africa; National and Kapodistrian University of Athens, GREECE

## Abstract

**Introduction:**

Little information is available on the efficacy and safety of the dual combination of ripivirine plus dolutegravir. This work aims at beginning to fill this gap.

**Methods:**

All HIV-1 infected subjects treated with ripivirine plus dolutegravir between October 2014 and September 2015 in eight Italian centres were included in an observational cohort. Data were collected at baseline and at weeks 4, 12, 24 and 48.

**Results:**

One hundred and thirty-two subjects were followed for a median of 24 months, mean 33 months. One subject discontinued the study drug at week 24 for headache, one for drug interaction and one died after week 24 of illicit drug abuse. The mean age was 51.8, females 31.7% and non-caucasians 10%. Fifty-seven (43.2%) had at least one failure in their treatment history. Reasons for switching were simplification (53.0%), toxicity (34.8%), drug interactions (n = 7), persistent low-level viremia (n = 4), non-adherence (n = 3) and viral failure (n = 2). Sixty patients (45.5%) had reverse transcriptase (RT) mutations and 69 (44,7%) had protease (PR) mutations. Sixteen had baseline viral replication, 27 had < 50 HIV-1 RNA copies/mL and in 89 (67.4%) no virus was detected (NVD, 0 copies/mL). At w4, 114 (86.4%) had NVD, 15 had 1 to 49 HIV-1 RNA copies/mL and 3 had 50 to 57 copies/mL. At week 24 one subject had viral rebound without mutations due to missed drug refill, 19 had 1 to 49 copies/mL, and 112 had NVD. All 132 subjects were tested at weeks 4 and 24. Of the 50 subjects who had a 48-week follow-up, one had a treatment interruption, four had 1 to 49 copies/mL and 45 had NVD. Among the entire population, one subject had low-level, one intermediate and 4 high-level resistance to rilpivirine: none failed by week 48. Mean serum creatinine increased by +0.1 mg/dL. During the follow-up one patient reported headache and insomnia.

**Conclusions:**

Ripivirine plus dolutegravir proved safe and effective in this cohort of non-naïve HIV-1 infected subjects.

## Introduction

HIV-1-infected subjects on antiretroviral therapy are now facing the perspective of an improved life expectancy [1], hence issues of proactively avoiding metabolic and organ damage are becoming crucial, and strategies of nucleoside-nucleotide sparing regimens are sought [2]. A particularly attractive option under study is the long-acting combination of an integrase strand transfer inhibitor (INSTI) plus a non-nucleoside reverse transcriptase inhibitor (NNRTI), the most metabolic-friendly antiretroviral drug classes [3]. There are two ongoing phase III randomized clinical trials, SWORD-1 [4] and SWORD-2 [5], for assessing the safety and efficacy of switching from stable antiretroviral regimens composed of two nucleoside-nucleotide reverse transcriptase inhibitors (N_t_RTIs) plus a protease inhibitor (PI), INSTI or NNRTI to a combination of dolutegravir (DTG) plus rilpivirine (RPV). Each study enrolled approximately 500 patients from 13 countries, focusing on women and subjects over 50 years of age. The primary endpoint of such studies is the proportion of patients with plasma HIV-1 RNA <50 copies/mL at week 48. Secondary endpoints include evaluation of the development of viral resistance, measurements of safety and tolerability, and changes in renal, bone and cardiovascular biomarkers. A third trial, named DORISS, by the University of Nantes, was withdrawn prior to enrolment [6]. Also, a fixed-dose oral formulation of the two drugs is being developed [7].

A phase 1, open-label, crossover study showed a modest increase in DTG and RPV AUC and C_max_ in 16 healthy male and female adults [8], who received 50 mg DTG alone every 24 h for 5 days followed by a ≥7-day washout, then 25 mg RPV alone every 24 h for 11 days, immediately followed by DTG plus RPV every 24 h for 5 days. Differences in duration of the three periods were based on half-lives and time to steady-state. Finally, Shionogi, the Japanese company that first developed DTG, has also performed an *in vitro* study showing not only synergy between the two compounds, but also the ability to fully suppress viral replication over 90 days of culture in MT-2 cells at concentrations exceeding the EC_50_ by four folds and to allow viral replication without selecting resistance at lower concentrations [9].

Despite this, published data on the clinical use of this combination is limited to the experience in 11 subjects described by Camelia Gubavu et al. within a group of 31 patients on various DTG-based dual regimens [10]. Separate data for each regimen are not given, rather the dual therapy group is considered as a whole. Only one subject had HIV RNA > 50 copies/mL after a mean observation of 50 weeks (range 30–74) and the mean eGFR decreased by 4.5 mL/min/1.73 m^2^. The aim of this study is to provide an initial set of data about the clinical use and safety of this combination in a cohort of unselected patients.

## Materials and Methods

All subjects who started DTG plus RPV between October, 1, 2014 and September, 30, 2015 were included in an observational multicenter cohort named TivEdO (Tivicay plus Edurant Observational Cohort). After approval by Ethics Committees, no further enrolment was allowed to discourage undue influence on clinical practice (i.e.: switching patients to DTG plus RPV or adding exams for research purposes rather than for clinical needs). Only clinical events, demographic data, CD4 cell counts, HIV-1 RNA, serum creatinine and urinary proteins were deemed relevant for this study. The *glomerular filtration rate* (e-GFR) was estimated at baseline and at follow-up according to the Chronic Kidney Disease–Epidemiology Collaboration (CKD-EPI) equation [11]. For the analysis, it was decided to consider not only the classical cut-off value of 50 copies/mL, but also to split values below such threshold between detectable (1–49 copies) and undetectable viremia (no virus detected, NVD = 0 copies). Also, data of therapeutic drug monitoring were included in the analysis, considering only those cases in which the physician had requested the test for clinical purposes. Participating subjects signed an informed consent, the study was approved by the Ethics’ Committee of the coordinating centre extended to all the Ethics’ Committees of involved centers.

✓ Comitato Etico Interaziendale Milano Area A.
✓ Comitato Etico delle Aziende Sanitarie dell’Umbria, Perugia.
✓ Comitato Etico Arnas Garibaldi, Catania.
✓ Comitato Etico Regionale Regione Liguria.
✓ Comitato Etico Sperimentazione Clinica Azienda Ospedaliera-Universitaria Careggi, Firenze.
✓ Comitato Etico dell'Università Cattolica del Sacro Cuore—Policlinico Universitario Agostino Gemelli, Roma
✓ Comitato Etico IRCCS Policlinico San Matteo, Pavia
✓ Comitato Etico della Asl to/2 di Torino

The study has been conducted according to the Good Clinical Practice (GCP) Guidelines.

The patients were controlled (visit and blood work) about 4 weeks after simplification to the study regimen, and subsequently at months 3, 6 and 12. For homogeneity no data were collected after week 48. For the toxicity analysis the Common Terminology Criteria for Adverse Events (CTCAE, Version 4.03, June 14, 2010) was considered [12]. Adverse clinical events and deaths were reported to the local Ethics Committees and authorities as required by law. Data were collected by the local investigators and periodically sent to the coordinating centre at ‘‘Luigi Sacco” Hospital, Milan. The follow-up for this presentation was censored on March, 31, 2016.

The statistical analysis of this study is limited to median values and ranges for the immunologic aspect. No statistical significance was assessed given the non homogeneity of the population.

## Results

**Fig 1:**
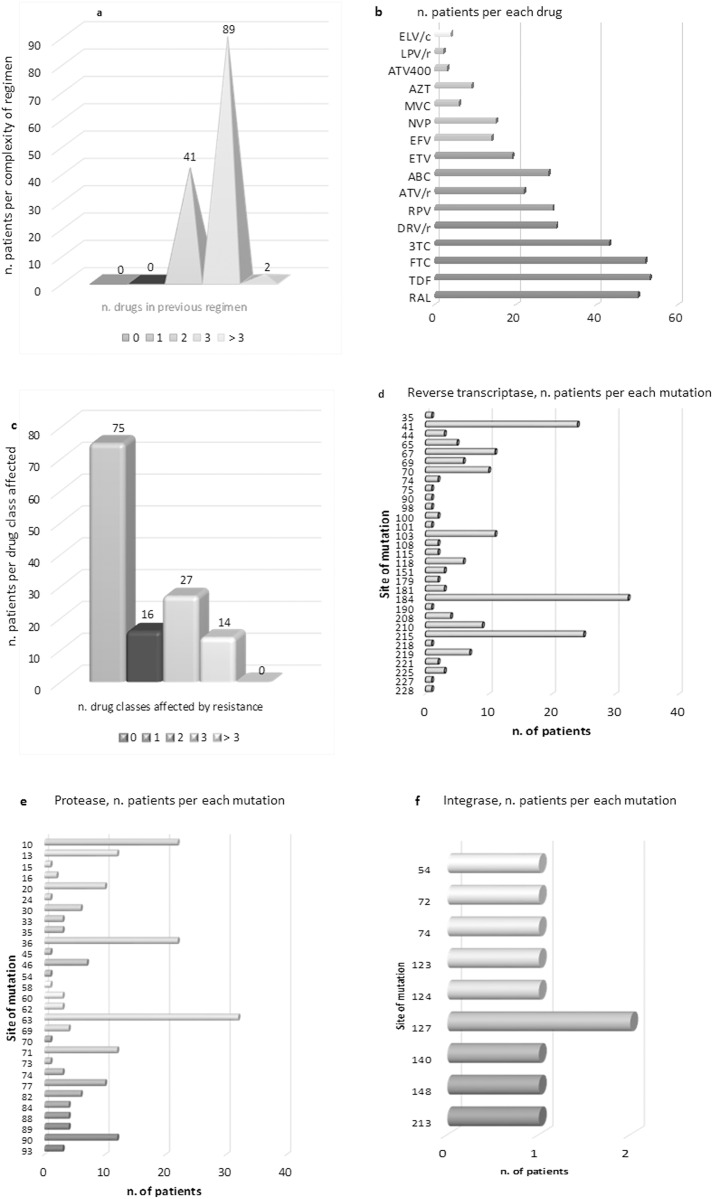
Patients’ baseline disposition towards antiretrovirals was based on: a) the complexity of the former regimen; b) the frequency of single antivirals in the former regimens of the population; c) the complexity of drug resistance at baseline; d), e), f) the frequency of single baseline mutations in the population by reverse transcriptase, protease and integrase regions, respectively. ELV/c = elvitegravir/cobicistat, AZT = zidovudine, LPV = lopinavir, /r = boosted with ritonavir, ATV = atazanavir, MVC = maraviroc, NVP = nevirapine, EFV = efavirenz, ETV = etravirine, ABC = abacavir, RPV = rilpivirine, DRV = darunavir, 3TC = lamivudine, FTC = emtricitabine, TDF = tenofovir, RAL = raltegravir.

One hundred and thirty-two subjects were enrolled in this cohort and followed for a median of 24 months, mean 33 months. The median age was 51.8 (range 29–77), females were 42 (31.7%), non-caucasians 13 (10%), and risks factors were balanced (29.5%, n = 39, drug abuse, 33.3%, n = 44, heterosexual intercourse and 35.6%, n = 47, male homosexual intercourse, plus 0.8%, n = 1 each, vertical and transfusional infection). Fifty-seven (43.2%) had had at least one failure with a previous antiretroviral regimen. The main reason for the switch was simplification (53.0%, n = 70), followed by toxicity (34.8%, n = 46: 9.8%, n = 13, osteopenia/osteoporosis, 9.1%, n = 12, hyperlipidemia, 5.3%, n = 7, gastrointestinal intolerance, 3.8, n = 5, cardiovascular problems, 3.0%, 4, glucose intolerance, 3.0%, 4, mental disturbances and 0.7%, 1, liver toxicity), drug-drug interactions with anti-HCV therapy (5.3%, n = 7), persistent low-level viremia (3.0%, n = 4), non-adherence (2.3%, n = 3) and viral failure (1.6%, n = 2). The complexity of the former regimen and the frequency of resistance mutations at baseline are reported in Fig 1.

One patient discontinued study drug at week 24 for headache, one for drug interaction and one died after week 24 from illicit drug abuse.

Sixty patients (45.5%) had reverse transcriptase (RT) mutations–of which 46 to NRTIs alone, one to NNRTIs alone and 13 to both, including six to RPV, 69 (44,7%) had protease (PR) mutations; one had full INSTI resistance but is, nevertheless, taking DTG twice daily, having no other choice due to drug interaction and resistance. Eight subjects (6.1%) never had a genotype for historical reasons.

**Fig 2:**
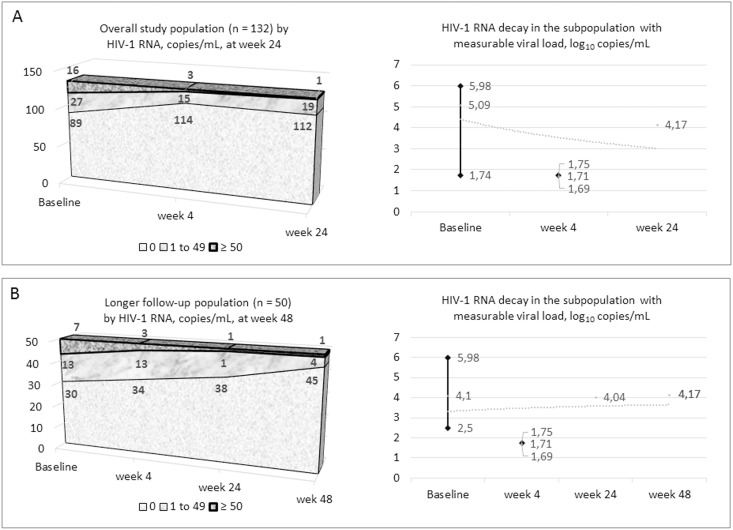
Virologic response in the global population (A) and in the subgroup that has reached week 48 (B). Proportion of viremia ranks over time, and median viral decay (range) and trend in viremic subjects.

The virologic baseline condition and outcomes are described in Fig 2.

The population who had viral load >50 copies/mL narrowed from baseline (n = 16), to week 4 (n = 3). At week 24 only one subject had viral rebound without mutations due to missed drug refill (11030 copies/mL). Of the 50 patients who had a 48-week follow-up, the above mentioned subject had another treatment interruption, with 14770 copies/mL, four had 1–49 copies/mL and 45 had NVD. The patient who had frequently interrupted therapy after baseline accumulated RT mutations 138Q and 181C, while the integrase gene was unaffected.

**Fig 3:**
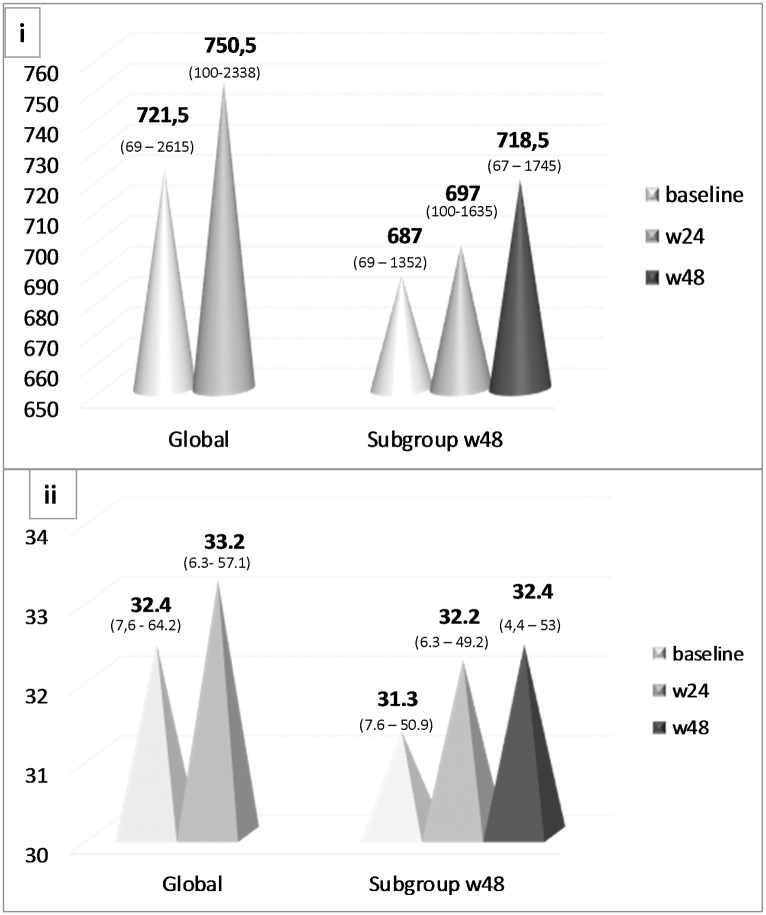
Absolute CD4+ T-cell count (i) and proportion of CD4+ T-cells (ii) in the global population and in the subgroup that reached week 48, median (range).

CD4+ T-cells absolute count and proportion increased over time both in the entire cohort and in the week 48 subgroup, as shown in Fig 3.

One subject had low-level resistance to rilpivirine, one intermediate and 4 high-level resistance (Stanford median score 50, range 15–70), but none failed, all having a 48-week follow-up.

Therefore, in this population no viral failures were observed in the short term, while the proportion of subjects with any detectable viremia decreased by 50%.

The median variation in serum creatinine was +0.1 (IQR + 0.23 to– 0.18, range + 0.47 to– 0.31) and the median eGFR decline was 10.3 ml/min (from 87.4 to 77.1). One subject had mild proteinuria at baseline that resolved spontaneously by week 24. Over the follow-up period, only one patient reported headache and insomnia, which lead to treatment discontinuation. Finally, the pharmacokinetic analysis of both drugs’ C_trough_ performed in 9 subjects revealed suboptimal concentrations of rilpivirine in 4 subjects and low concentrations in general(<20–38 ng/mL), while dolutegravir median level was 445 ng/mL (range 334–1112).

Furthermore, comparing the cost of the former treatment line with the study regimen, there is no additional economic burden, rather the dual therapy saves € 3.90 per day per patient (22.69 vs 26.59 €/day).

## Discussion

We describe the experience of several Italian physicians who switched their patients to RPV plus DTG exclusively for clinical reasons.

Apart from the perspective of future long-acting parenteral therapies combining INSTIs with RPV [3], this combination provides a simplified option for those subjects who have developed resistance to nucleoside analogues or who have a history of suboptimal therapies (i.e. two nucleosides) and cannot benefit from single-tablet regimens, as well as for those subjects who need metabolic-friendly drugs, having important comorbidities or being predisposed to develop toxicity.

The TivEdO observational study showed that dual regimen therapy with DTG plus RPV was safe and effective, confirming what was described by Gubavu et al [10] and by Sued et al [13], controlling viral replication in all the subjects who took the therapy and fully suppressing viremia in 81% of those evaluated at week 24 and in 90.9% of the subgroup who had a 48-week follow-up. Prudence is required in drawing conclusions, as randomized clinical trials still have to confirm such data. Moreover, in the subject who had several treatment interruptions, the integrase gene resulted unaffected while two new resistance-associated mutations towards NNRTIs appeared on a background of dual-class resistance.

Also, the particularly small pill size was very appreciated by the patients.

The regimen was overall well tolerated and just one subject discontinued it at week 24 for headache. Our experience did not reveal low tolerability of DTG as recently described by a group of Dutch colleagues [14]. Our population was numerically smaller, but wide enough to detect a problem of that size, and the observation period was comparable. Maybe the fact that 40.1% of the subjects in our study came from another INSTI created some selection of class-related side effects (such as headache and dizziness), and our tolerability data described only the dual combination studied.

The drug-drug interaction that led to discontinuation was between RPV and proton pump inhibitors, irreplaceable in a subject affected by gastro-esophageal reflux.

The switch did not increase the cost of therapy, although this was not a driver towards it.

Clearly, this observational study has many limitations: first of all, the only two inclusion criteria were being HIV-1 antibody positive and having started DTG plus RPV; second, the population was rather heterogeneous in (i.e.: wide CD4+ T-cell range, baseline viremia, reasons for switching). Finally, we only took a small group of parameters of safety into consideration, besides attainment and maintenance of virologic suppression.

## Conclusions

In this setting no cases of virological failure were observed in patients who adhered to the regimen DTV plus RPV; indeed, the only patient who had virological failure, accumulating also mutations to NNRTIs, was frequently off therapy due to problems with drug refill. The combination was well tolerated, with only one drop-out due to tolerability issues. Randomized clinical trials will make the potential of this attractive regimen more evident.

## Supporting Information

S1 Appendix Authors’ contribution to the work. (PPT)

S2 Appendix List of the Ethics’ Committees and respective centres. (PPT)

S3 Appendix Database for Data Sharing. (XLSX)

S4 Appendix Protocollo TivEdO, Version 1.0, July, 14, 2015. (PDF)
