# Supplementary material for: Switch to Dolutegravir plus Rilpivirine Dual Therapy in cART-Experienced Subjects: An Observational Cohort
Source: PLoS One. 2016 Oct 14;11(10):e0164753. doi: 10.1371/journal.pone.0164753 (PMC5065232; doi:10.1371/journal.pone.0164753)
Supplement: S1 Appendix — (PPT) [file pone.0164753.s001.ppt]

## Slide 1
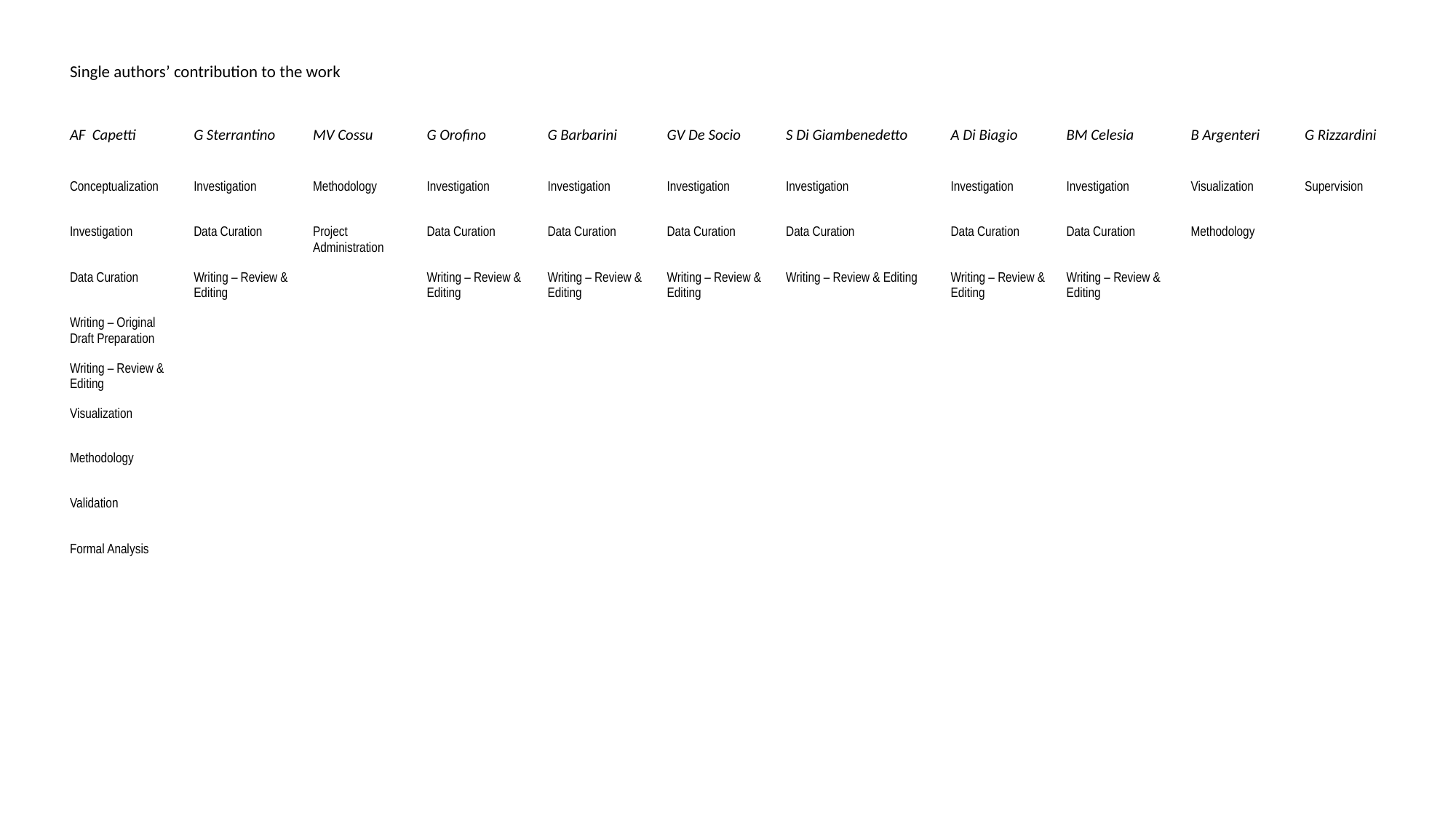

Single authors’ contribution to the work
| AF Capetti | G Sterrantino | MV Cossu | G Orofino | G Barbarini | GV De Socio | S Di Giambenedetto | A Di Biagio | BM Celesia | B Argenteri | G Rizzardini |
| --- | --- | --- | --- | --- | --- | --- | --- | --- | --- | --- |
| Conceptualization | Investigation | Methodology | Investigation | Investigation | Investigation | Investigation | Investigation | Investigation | Visualization | Supervision |
| Investigation | Data Curation | Project Administration | Data Curation | Data Curation | Data Curation | Data Curation | Data Curation | Data Curation | Methodology | |
| Data Curation | Writing – Review & Editing | | Writing – Review & Editing | Writing – Review & Editing | Writing – Review & Editing | Writing – Review & Editing | Writing – Review & Editing | Writing – Review & Editing | | |
| Writing – Original Draft Preparation | | | | | | | | | | |
| Writing – Review & Editing | | | | | | | | | | |
| Visualization | | | | | | | | | | |
| Methodology | | | | | | | | | | |
| Validation | | | | | | | | | | |
| Formal Analysis | | | | | | | | | | |
