# Supplementary material for: Switch to Dolutegravir plus Rilpivirine Dual Therapy in cART-Experienced Subjects: An Observational Cohort
Source: PLoS One. 2016 Oct 14;11(10):e0164753. doi: 10.1371/journal.pone.0164753 (PMC5065232; doi:10.1371/journal.pone.0164753)
Supplement: S2 Appendix — (PPT) [file pone.0164753.s002.ppt]

## Slide 1
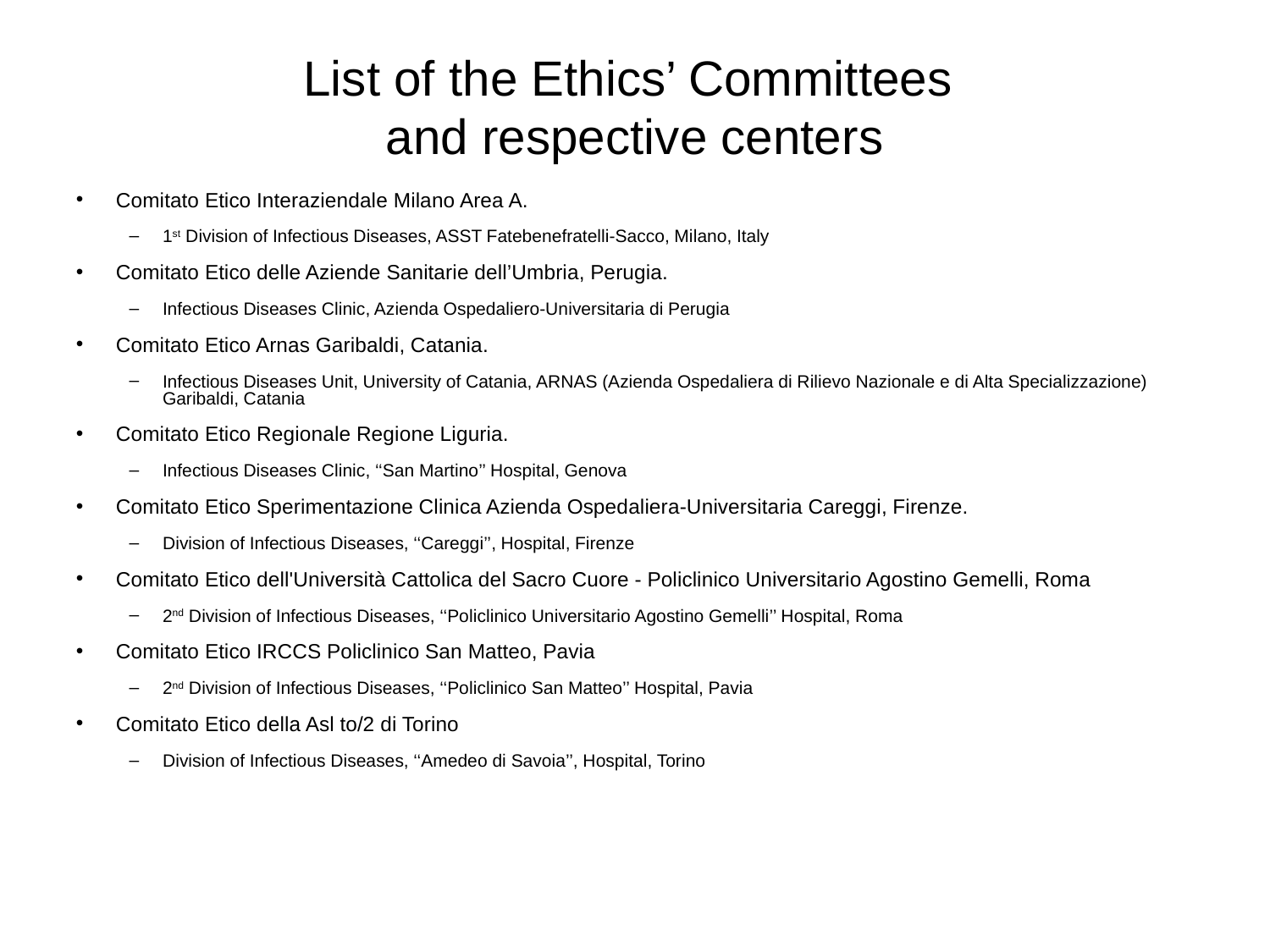

# List of the Ethics’ Committees and respective centers
Comitato Etico Interaziendale Milano Area A.
1st Division of Infectious Diseases, ASST Fatebenefratelli-Sacco, Milano, Italy
Comitato Etico delle Aziende Sanitarie dell’Umbria, Perugia.
Infectious Diseases Clinic, Azienda Ospedaliero-Universitaria di Perugia
Comitato Etico Arnas Garibaldi, Catania.
Infectious Diseases Unit, University of Catania, ARNAS (Azienda Ospedaliera di Rilievo Nazionale e di Alta Specializzazione) Garibaldi, Catania
Comitato Etico Regionale Regione Liguria.
Infectious Diseases Clinic, ‘‘San Martino’’ Hospital, Genova
Comitato Etico Sperimentazione Clinica Azienda Ospedaliera-Universitaria Careggi, Firenze.
Division of Infectious Diseases, ‘‘Careggi’’, Hospital, Firenze
Comitato Etico dell'Università Cattolica del Sacro Cuore - Policlinico Universitario Agostino Gemelli, Roma
2nd Division of Infectious Diseases, ‘‘Policlinico Universitario Agostino Gemelli’’ Hospital, Roma
Comitato Etico IRCCS Policlinico San Matteo, Pavia
2nd Division of Infectious Diseases, ‘‘Policlinico San Matteo’’ Hospital, Pavia
Comitato Etico della Asl to/2 di Torino
Division of Infectious Diseases, ‘‘Amedeo di Savoia’’, Hospital, Torino
