# Supplementary material for: Switch to Dolutegravir plus Rilpivirine Dual Therapy in cART-Experienced Subjects: An Observational Cohort
Source: PLoS One. 2016 Oct 14;11(10):e0164753. doi: 10.1371/journal.pone.0164753 (PMC5065232; doi:10.1371/journal.pone.0164753)
Supplement: S4 Appendix — (PDF) [file pone.0164753.s004.pdf]

Ospedale Luigi Sacco
AZIENDA OSPEDALIERA - POLO UNIVERSITARIO

Sistema Sanitario Regione Lombardia

Studio TIVedo

STUDIO OSSERVAZIONALE, RETROSPETTIVO, MULTICENTRICO PER VALUTARE LA SICUREZZA E L'EFFICACIA DEL PASSAGGIO AD UN REGIME ANTIRETROVIRALE COSTITUITO DA DOLUTEGRAVIR E RILPIVIRINA

Tipologiadocumento	Protocollo studio clinico
Tipologia studio	Osservazionale
Status Documento:	Versione Finale 1.0
Data.	14 Luglio 2015
Numero di pagine:	7

Proprietà dell'Ospedale Luigi Sacco
Il presente documento non può essere usato, divulgato
o pubblicato senza l'autorizzazione dell'Ospedale Luigi Sacco

Promotore:

Dott. Amedeo Capetti
(Responsabile del Progetto)

Firma

29-07-2015
Data

Sperimentatore:

Dott. Amedeo Capetti
(Principal Investigator)

Firma

29-07-2015
Data

1. INTRODUZIONE

1.1. *Farmacologia di* Dolutegravir

Dolutegravir (DTG) è il più recente inibitore dell'integrasi (INSTI, integrase strand transfer inhibitor) immesso in commercio, attivo su HIV-1 e su HIV-2 [1].

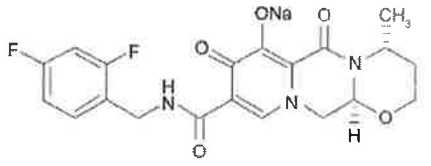

DTG è rapidamente assorbito a livello gastrointestinale (T_{max}, tempo al picco plasmatico = 2,1 h) e l'assunzione con il cibo ne aumenta la biodisponibilità (dal 31 al 66% in più) e la T_{max} (fino a 5 h). Il suo legame con le proteine plasmatiche è del 99%, ed è principalmente metabolizzato da UGT 1A1, e in minor misura da 1A3 e da 1A9 e da CYP3A4. L'emivita è di circa 12 h, con modesta variabilità interindividuale. Non è necessario assumere DTG a stomaco pieno se il virus HIV è sensibile, mentre in caso di mutazioni correlate a resistenza questa misura può essere utile.

Non sono state evidenziate differenze nella farmacocinetica di DTG per età, razza o sesso. I soggetti con cirrosi moderata (Child-Pugh B) hanno livelli di DTG sierico libero 1.5-2 volte superiori al normale ma non è necessario un aggiustamento, mentre non vi sono dati nella cirrosi grave (Child-Pugh C).

Il passaggio di DTG nel liquor e nel compartimento genitale è ottimale [2,3,4].

DTG presenta interazioni farmacologiche significative con etravirina, nevirapina, dofetilide, carbamazepina, fenitoina e fenobarbital, che dovrebbero essere evitati, rifampicina, efavirenz, fosamprenavir e tipranavir, che richiedono un raddoppio del dosaggio di DTG, e con metformina , che richiede un più attento monitoraggio.

1.2. Barriera genetica alla resistenza

DTG ha mostrato di possedere una barriera genetica superiore rispetto agli altri INSTI commerciali (raltegravir - RAL, elvitegravir - EVG), in parte grazie alla sua lunghissima emivita di dissociazione dal sito di azione (70 h) [5]. DTG è attivo contro i ceppi di HIV-1 –resistenti a RAL e a EVG con le più comuni mutazioni associate a resistenza (RAMs), fra cui Y143C/H/R, N155H, and Q148H/K/R in assenza di mutazioni secondarie [6]. La selezione *in vitro* delle RAMs E92Q, G118R, S153FY, G193E, o R263K riduceva la sensibilità del virus di circa 4 volte. Ceppi contenenti la mutazione Q148HR, accumulando ulteriori mutazioni (T97A, E138K, G140S, e M154I) in passaggi seriali diovenivano 13-46-volte meno sensibili a DTG. L'associazione di Q148HR e G140S portava allo sviluppo di ulteriori RAMs (L74M, E92Q, N155H) nei successivi passaggi.

Negli studi clinici finora condotti su soggetti naïve alla terapia antiretrovirale (SPRING-1, SPRING-2, SINGLE, FLAMINGO) si è osservata la selezione di mutazioni di resistenza, fino a 96 settimane [7,8,9,10].

Negli studi sui soggetti che avevano fallito regimi di terapia antiretrovirale ma INSTI-naïve < 1% ha selezionato RAMs verso DTG; invece, il 45% dei pazienti che avevano fallito regimi contenenti INSTI sviluppando RAMS verso RAL o EVG selezionavano ulteriori mutazioni durante la terapia con DTG [11,12]. Gli studi VIKING-2 e -3 dimostrano che è necessario acquisire multiple INSTI RAMs per ridurre significativamente l'efficacia di DTG. I soggetti falliti ad INSTI con la mutazione Q148HR e ≥ 2 RAMs

aggiuntive (L74IM, E138ADKT, G140A/S, Y143HR, E157Q, G163EKQRS, G193ER) mostravano la massima perdita di efficacia da parte di DTG, e solo il 18% di questi sopprimeva HIV-1 RNA sotto 50 copie/mL dopo 24 settimane di terapia [13]. La combinazione di mutazioni più comune era nei siti Q148+ G140+ E138. La presenza di sostituzioni in N155 o Y143 senza Q148 o di Q148HR + G140AS senza altre RAMs consentiva ancora al 56%–80% dei pazienti di sopprimere il virus. Si è recentemente scoperto che le mutazioni G118R e F121Y conferiscono al virus una riduzione di sensibilità a DTG maggiore di 100 [14].

Dati di sorveglianza nazionale US suggeriscono che il 12% dei soggetti che presenta virus resistente a RAL o a EVG abbia anche elevata resistenza a DTG [15].

Efficacia negli studi clinici

Grazie all'efficacia particolare dell'intera classe ed alle sue peculiari caratteristiche farmacodinamiche DTG ha dimostrato negli studi clinici sui soggetti naive la superiorità nei confronti di efavirenz e darunavir/ritonavir e la non inferiorità nei confronti di raltegravir. Nei soggetti che avevano già presentato fallimenti a precedenti terapie ha mostrato la superiorità nei confronti di raltegravir ed eccellenti percentuali di successo in monoterapia funzionale o in soggetti con INSTI-RAMs. DTG si è pertanto dimostrato il più efficace singolo farmaco antiretrovirale verso HIV-1, con una barriera genetica paragonabile ai bPIs.

Tab. 1 Studi clinici condotti e conclusi su dolutegravir

Studio	***End-point primario***	***Sicurezza***
**Naive**		
SPRING-2[8]	Noninferiorità *vs* RAL nel sopprimere la viremia a < 50 copie/mL a 48 e 96 settimane	Simile fra RAL e DTG
SINGLE[9]	Superiorità *vs* tenofovir/emtricitabine/efavirenz nel sopprimere la viremia a < 50 copie/mL a 48 e 96 settimane	Eventi avversi più comuni in tenofovir/emtricitabina/efavirenz (43% *vs* 66%)
FLAMINGO[10]	Superiorità vs regimi basati su darunavir/ritonavir nel sopprimere la viremia a < 50 copie/mL a 48 settimane	Livelli di lipidi e interruzioni dello studio più frequenti nel gruppo in darunavir/ritonavir
**Pre-trattati**		
SAILING[11]	Superiorità vs raltegravir nel sopprimere la viremia a < 50 copie/mL a 48 settimane	Simile fra RAL e DTG
VIKING[12]	HIV-1 RNA < 400 copie/mL al giorno 11<br>In monoterapia funzionale con DTG nel 78% dei soggetti trattati con DTG 50 mg OD e nel 96% dei trattati con 50 mg BID	Non eventi gravi o interruzioni correlate a DTG
VIKING-3[13]	L'aggiunta di DTG al regime fallimentare permette al 69% dei soggetti di sopprimere la viremia	Cinque interruzioni di studio correlate a DTG; tre epatiti, una colelitiasi, aed una sindrome con rash, prurito e parestesie. Un caso di sincope ed uno di HSR
IMPAACT-P1093[16,17]	Coorte 1 (età 6–12 anni): soppressione della viremia a < 400 copie/mL nell'81.8% ed a <50 copie/mL nel 63.6%<br>Coorte 2 (età 12–18 anni): soppressione della viremia a < 400 copie/mL nel 73.9% ed a <50 copie/mL nel 60.9%	Non eventi gravi o interruzioni correlate a DTG

Grazie a queste osservazioni, analogamente ai bPIs DTG sarà valutato sia in studi di monoterapia[18] sia di duplice terapia.

Tab. 2 Studi clinici pianificati e di prossima apertura su dolutegravir in mono- o duplice terapia

Companion Drug	Study
3TC	**Dolutegravir-Lamivudine as Dual Therapy in Naive HIV-Infected Patients: A Pilot Study (PADDLE)**[19]<br>*Ongoing, but not recruiting participants*<br>Sponsor: The Huesped Foundation - Collaborator: ViiV Healthcare<br>Principal Investigator: Dr Pedro Cahn, The Huesped Foundation, Buenos Ayres
Maraviroc	**Switch to Maraviroc + Integrase Inhibitor**<br>*This study is currently recruiting participants*<br>Sponsor: University of Maryland - Principal Investrigator: Dr David J Riedel, University of Maryland
Rilpivirine	**Relative Oral Bioavailability Study of Different Fixed Dose Combinations of Dolutegravir and Rilpivirine in Healthy Subjects**[20]<br>*This study is currently recruiting participants*<br>Sponsor: ViiV Healthcare - Collaborators: Janssen Pharmaceuticals, GlaxoSmithKline<br>**Dolutegravir + Rilpivirine Switch Study (DORISS)**[21]<br>*This study is not yet open for participant recruitment*<br>Sponsor: Nantes University Hospital<br>Principal investigators: Bruno Hoen, Point à Pitre University Hospital & François Raffi, Nantes University Hospital<br>**Regimen Switch to Dolutegravir + Rilpivirine From Current Antiretroviral Regimen in Human Immunodeficiency Virus Type 1 Infected and Virologically Suppressed Adults (SWORD-1)**[22]<br>*This study is currently recruiting participants*<br>Sponsor: ViiV Healthcare - Collaborator: Janssen, GlaxoSmithKline (GSK)<br>**Regimen Switch to Dolutegravir + Rilpivirine From Current Antiretroviral Regimen in Human Immunodeficiency Virus Type 1 Infected and Virologically Suppressed Adults (SWORD-2)**[23]<br>*This study is not yet open for participant recruitment*<br>Sponsor: ViiV Healthcare - Collaborator: Janssen, GlaxoSmithKline (GSK)

Dalla pubblicazione di dolutegravir in Gazzetta Ufficiale (18 Ottobre 2014), e dalla sua effettiva disponibilità presso i centri di Malattie Infettive italiani, si è osservato che le caratteristiche singolari del farmaco ne permettevano l'utilizzo anche oltre gli schemi degli studi registrativi, consentendo di semplificare e rendere più economici, ad esempio, svariati regimi complessi nei soggetti multifalliti, oppure, in altri casi, in particolare in associazione a rilpivirina, a superare complessi problemi di interazioni farmacologiche.

Per tale motivo, avendo osservato una diffusione di regimi 'non convenzionali', abbiamo deciso di valutarli in un'analisi retrospettiva.

2. OBIETTIVI

2.1. Obiettivo primario

Valutare, nella normale pratica clinica, la tollerabilità del passaggio alla combinazione in studio

2.2. Obiettivo secondario

- Valutare l'efficacia del passaggio alla terapia in studio in termine di percentuale di soggetti con:

a) viremia inferiore alla soglia di misurazione, ma rilevabile

b) viremia non rilevabile a sei mesi dal passaggio alla nuova combinazione

3. DISEGNO DELLO STUDIO

Questo è uno studio osservazionale, multicentrico, di coorte, retrospettivo, che ha lo scopo di raccogliere i dati sulla tollerabilità e sulla risposta al trattamento del passaggio ad una terapia con Dolutegravir/Rilpivirina.

Saranno invitati a partecipare allo studio circa 15 centri di malattie infettive.

I pazienti saranno osservati (in modo prospettico e/o retrospettivo) dall'inizio della durata del trattamento per quanto riguarda l'analisi di tollerabilità e per 6 mesi per quanto riguarda l'analisi di risposta al trattamento.

Materiali e metodi

Saranno analizzati i database delle Unità di Malattie Infettive aderenti al progetto a partire dal Novembre 2014. Saranno inclusi tutti i soggetti che in qualunque momento e per qualunque durata avranno assunto tale combinazione, per l'analisi di tollerabilità. Per l'analisi di efficacia invece saranno inclusi solo i soggetti arrivati a 6 mesi di osservazione dal momento in cui hanno iniziato una delle suddette combinazioni, indipendentemente dal fatto che abbiano proseguito la terapia o che l'abbiano sospesa.

L'analisi riguarderà solo gli esami eseguiti di routine, ossia profilo lipidico, creatininemia, es. urine, HIV-1 RNA, CD4, la presenza di RAMs al basale ed all'eventuale fallimento, e, nei soggetti per cui il curante lo abbia ritenuto necessario, l'analisi della C_{min} dei farmaci antiretrovirali.

Criteri di inclusione

- Infezione da HIV-1 documentata (almeno una viremia rilevabile o HIV Ab pos)
- Uomini o donne che abbiano compiuto almeno 18 anni di età dall'inizio del trattamento
- Soggetti che abbiano iniziato un regime antiretrovirale composto da Tivicay 50 mg + Edurant 25 mg ogni 24 ore almeno 6 mesi prima dell'arruolamento, indipendentemente dal fatto che la terapia sia ancora in corso.
- Soggetti in grado di comprendere e firmare un consenso informato

4. ANALISI STATISTICA

Poiché non ci sono dati in letteratura, le dimensioni del campione sono state stabilite in base ai criteri di fattibilità, da cui è emerso il volume di pazienti arruolabili attualmente gestiti dai centri coinvolti nello studio. E' ragionevole stimare l'inclusione di circa 30 pazienti totali con le caratteristiche descritte nei criteri di inclusione.

Per tale motivo la statistica si limiterà ad una analisi descrittiva (mediana, range) di tutti i dati raccolti, includendo tutti i pazienti che abbiano assunto il regime in studio per almeno una somministrazione.

Considerazioni finali

La rilevanza di tale studio dipende dal fatto che non esistono attualmente dati clinici riguardanti efficacia e tollerabilità di questo regime antiretrovirale.

---

[1] European Medicines Agency. Product information for Tivicay, 2014. Available from: http://www.ema.europa.eu/docs/en_GB/document_library/EPAR_-_Product_Information/human/002753/WC500160680.pdf. Accessed November 6, 2014.
[2] Letendre SL, Mills AM, Tashima KT, et al. ING116070: A study of the pharmacokinetics and antiviral activity of dolutegravir in cerebrospinal fluid in HIV-1-infected, ART-naïve subjects. Clin Infect Dis. 2014;59:1032–1037.
[3] Greener BN, Patterson KB, Prince HM, et al. Dolutegravir pharmacokinetics in the genital tract and colorectum of HIV-negative men after single and multiple dosing. J Acquir Immune Defic Syndr. 2013;64:39–44.
[4] Adams JL, Patterson KB, Prince HM, et al. Single and multiple dose pharmacokinetics of dolutegravir in the genital tract of HIV-negative women. Antivir Ther. 2013;18:1005–1013.
[5] DeAnda F, Hightower KE, Nolte RT, et al. Dolutegravir interactions with HIV-1 Integrase-DNA: structural rationale for drug resistance and dissociation kinetics. PLoS One. 2013;8:e77448.
[6] Canducci F, Ceresola ER, Boeri E, et al. Cross-resistance profile of the novel Integrase inhibitor dolutegravir (S/GSK1349572) using clonal viral variants selected in patients failing raltegravir. J Infect Dis. 2011;204:1811–1815.
[7] van Lunzen J, Maggiolo F, Arribas JR, et al. Once-daily dolutegravir (S/GSK1349572) in combination therapy in antiretroviral-naïve adults with HIV: planned interim 48 week results from SPRING-1, a dose-ranging, randomised, phase 2b trial. Lancet Infect Dis. 2012;12:111-118.
[8] Raffi F, Rachlis A, Stellbrink HJ, et al. Once-daily dolutegravir versus raltegravir in antiretroviral-naive adults with HIV-1 infection: 48 week results from the randomised, double-blind, non-inferiority SPRING-2 study. Lancet. 2013;381:735–743.
[9] Walmsley SL, Antela A, Clumeck N, et al. Dolutegravir plus abacavir-lamivudine for the treatment of HIV-1 infection. N Eng J Med. 2013;369:1807–1818.
[10] Clotet B, Feinberg J, van Lunzen J, et al. Once-daily dolutegravir versus darunavir plus ritonavir in antiretroviral-naïve adults with HIV-1 infection (FLAMINGO): 48 week results from the randomised open-label phase 3b study. Lancet. 2014;383;2222–2231.
[11] Cahn P, Pozniak AL, Mingrone H, et al. Dolutegravir versus raltegravir in antiretroviral-experienced, integrase-inhibitor-naïve adults with HIV: week 48 results from the randomised, double-blind, non-inferiority SAILING study. Lancet. 2013;382:700–708.
[12] Eron JJ, Bonaventua C, Durant J, et al. Safety and efficacy of dolutegravir in treatment-experienced subjects with raltegravir-resistant HIV type 1 infection: 24-week results of the VIKING study. J Infect Dis. 2013;207:740–748.
[13] Castagna A, Maggiolo F, Penco G, et al. Dolutegravir in antiretroviral-experienced patients with raltegravir- and/or elvitegravir-resistant HIV-1: 24-week results of the phase 3 VIKING-3 Study. J Infect Dis. 2014;210:354–362.
[14] Malet I, Gimferrer Arriaga L, Artese A, et al. New raltegravir resistance pathways induce broad cross-resistance to all currently used integrase inhibitors. J Antimicrob Chemother. 2014;69:2118–2122.
[15] Hurt CB, Sebastian J, Hick CB, Eron JJ. Resistance to HIV integrase strand transfer inhibitors among clinical specimens in the United States, 2009–2012. Clin Infect Dis. 2014;58:423–431.
[16] Viani RM, Alvero C, Fenton T, et al. Safety, pharmacokintetics, and efficacy of dolutegravir in treatment experienced HIV positive children. Poster presented at: 21st Conference on Retroviruses and Opportunistic Infections; March 3–6, 2014; Boston, MA.
[17] Viani RM, Alvero C, Fenton T, et al. Safety and efficacy of dolutegravir in HIV treatment-experienced adolescents: 48-week results. Poster presented at: 21st Conference on Retroviruses and Opportunistic Infections; March 3–6, 2014; Boston, MA.
[18] https://clinicaltrials.gov/ct2/show/NCT02401828
[19] https://clinicaltrials.gov/ct2/show/NCT02211482
[20] https://clinicaltrials.gov/ct2/show/NCT02373930
[21] https://clinicaltrials.gov/ct2/show/NCT02069834
[22] https://clinicaltrials.gov/ct2/show/NCT02429791
[23] https://clinicaltrials.gov/ct2/show/NCT02422797
